# Stable time patterns of railway suicides in Germany: comparative analysis of 7,187 cases across two observation periods (1995–1998; 2005–2008)

**DOI:** 10.1186/1471-2458-14-124

**Published:** 2014-02-06

**Authors:** Karoline Lukaschek, Jens Baumert, Natalia Erazo, Karl-Heinz Ladwig

**Affiliations:** 1 Institute of Epidemiology II, Helmholtz Zentrum München, German Research Centre for Environmental Health, Ingolstädter Landstr 1, Neuherberg 85764, Germany; 2 Department for Psychosomatic Medicine and Psychotherapy, Klinikum rechts der Isar, Technische Universität München, Munich, Germany

**Keywords:** Railway suicide, Time patterns, Gatekeeper programmes, Railway suicide prevention, Public Health

## Abstract

**Background:**

The majority of fatalities on the European Union (EU) railways are suicides, representing about 60% of all railway fatalities. The aim of this study was to compare time patterns of suicidal behaviour on railway tracks in Germany between two observation periods (1995–1998 and 2005–2008) in order to investigate their stability and value in railway suicide prevention.

**Methods:**

Cases were derived from the National Central Registry of person accidents on the German railway network (STABAG). The association of daytime, weekday and month with the mean number of suicides was analysed applying linear regression. Potential differences by observation period were assessed by adding observation period and the respective interaction terms into the linear regression. A 95% confidence interval for the mean number of suicides was computed using the t distribution.

**Results:**

A total of 7,187 railway suicides were recorded within both periods: 4,102 (57%) in the first period (1995–1998) and 3,085 (43%) in the second (2005–2008). The number of railway suicides was highest on Mondays and Tuesdays in the first period with an average of 3.2 and 3.5 events and of 2.6 events on both days in the second period. In both periods, railway suicides were more common between 6:00 am and noon, and between 6:00 pm and midnight. Seasonality was only prominent in the period 1995–1998.

**Conclusions:**

Over the course of two observation periods, the weekday and circadian patterns of railway suicides remained stable. Therefore, these patterns should be an integral part of railway suicide preventive measures, e.g. gatekeeper training courses.

## Background

Railway suicides represent only a minority of all suicides and the extent to which they contribute to total suicide mortality varies considerably among countries, e.g. Germany 7%, Netherlands 11.5% [1-4]. Nevertheless, the majority of fatalities on the EU railways are suicides, representing over 60% (N = 2429) of all railway fatalities [5]. The immense human and economic loss due to railway suicides calls for innovative preventive measures. Technical measures, such as restricting access, e.g. by installing physical barriers in strategic places [6-9], or the installation of blue lights on train platforms in Japan [10], are proving to be effective. Non-technical approaches, such as awareness programmes or gatekeeper training courses [3,9,11-13], are promising. An integral part of such courses is to recognize suicidal behavioural patterns which can help to identify potential railway suicide victims [14,15] and to be alert of specific high risk time windows of railway suicide (e.g. weekdays, daytime). So far, studies on railway suicides have revealed a frequency peak in April and September [16,17]. Regarding the weekly distribution, studies indicate a peak of suicide numbers at the beginning of the week and a low on weekends [17,18]. As for circadian patterns, previous analyses found a bimodal pattern with a morning peak between 9:00 am and noon, and an evening peak between 6:00 pm and 9:00 pm [16,17]. Notably, the peaks of the summer half year (April to September) compared to those in the winter half year (October to March) were clearly shifting in correspondence to the changing time of sunrise and sunset [17]. The knowledge of high-risk time windows can contribute to the development of effective approaches to prevention, if the underlying patterns were reliable. The investigation of temporal patterns and their stability over time is an important step in railway suicide prevention.

Thus, the aim of the study was to compare time patterns of suicidal behaviour on railway tracks in Germany between two observation periods (1995–1998 and 2005–2008) in order to investigate underlying principles of temporal patterns and to strengthen their usefulness in railway suicide preventive measures such as gatekeeper training courses.

## Method

### Study design and sample

Railway suicide cases were derived from the National Central Registry of all person accidents on the German railway net (STABAG) between 1995 and 1998 (first observation period) and between 2005 and 2008 (second observation period), satisfying the operational definition of an act of suicidal behaviour according to the International Classification of Diseases 10th revision (ICD-10) category “intentional self-harm by jumping or lying in front of a moving object” (code X81). Access to STABAG was possible by an internal cooperation with the German railway company Deutsche Bahn (DB).

The data for the present study were collected on a routine basis and particulars about personal or factual circumstances of a defined or definable natural person were factually anonymized. Thus, according to the German Federal Data Protection Act [19], no ethics approval was necessary.

Misclassifications were unlikely as the local police and the local coroner investigate every unnatural death by law. STABAG also contains information about the exact time of the incidence. In order to analyse the circadian distribution patterns, the day was divided into four periods of 6 h, beginning at midnight. In order to analyse the seasonal distribution patterns, the year was divided into its 12 months.

### Defining the observation periods

The rationale behind defining the two four-year time periods (1995–1998 and 2005–2008) was based upon the time point of the implementation of a German railway suicide prevention programme in 2002. We assumed that the potential effects of the measures of this prevention programme on the frequency of railway suicides are assessable only after some time delay; thus, we decided to choose a time period starting three years after the implementation which covers four years (aiming to obtain a sufficient number of observations). Analogously, we chose a control time period ending three years before the implementation of the prevention programme and covering also four years. The time frames were defined before any analyses were performed.

### Statistical analyses

The association of daytime, weekday and month with the mean number of railway suicides was analysed by means of linear regression. Potential differences in these associations by observation period were assessed by adding the observation period and the respective interaction terms into the regression. A 95% confidence interval (95% CI) for the mean number of railway suicides was computed using the t distribution.

For all statistical analyses, a p value less than 0.05 was considered to be statistically significant. All evaluations were performed with the statistical software package SAS Version 9.2. The analysis and the description in this article follow the STROBE guidelines for observational studies [20].

## Results

### Overall

A total of 7,187 railway suicides were recorded within both periods: 4,102 (57%) in the first period (1995–1998) and 3,085 (43%) in the second (2005–2008).

### Weekday patterns

**Figure 1:**
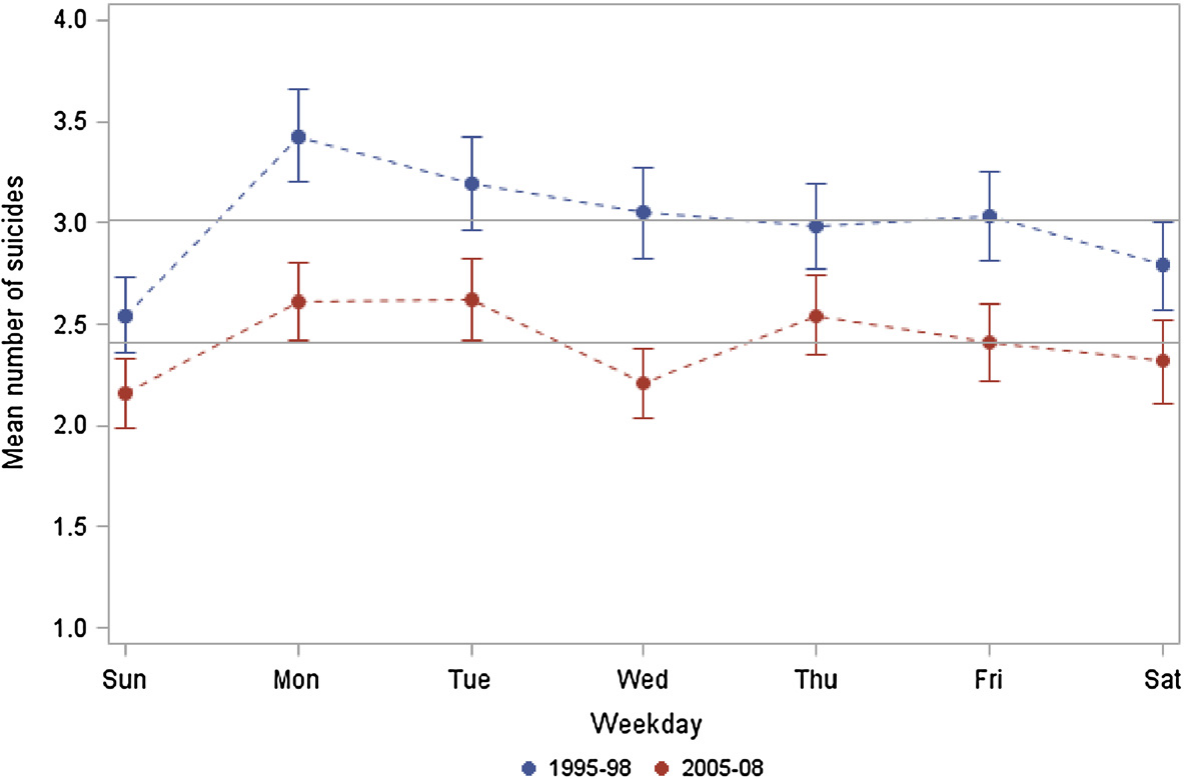
**Mean number of railway suicides per weekday (blue upper line: 1995–1998, red lower line: 2005–2008) with 95% confidence intervals.** The horizontal lines indicate the expected numbers in each period.

As shown in Figure 1, the distribution of railway suicides follows a similar pattern in both observation periods (p value for period effect 0.171), although the total number of railway suicides was lower in the second period. Looking at each period separately, the mean number of railway suicides differed significantly by weekday (p values < 0.001) and was highest on Mondays and Tuesdays with 3.5 and 3.2 events in the first period and 2.6 events on both days in the second period.

### Circadian patterns

**Figure 2:**
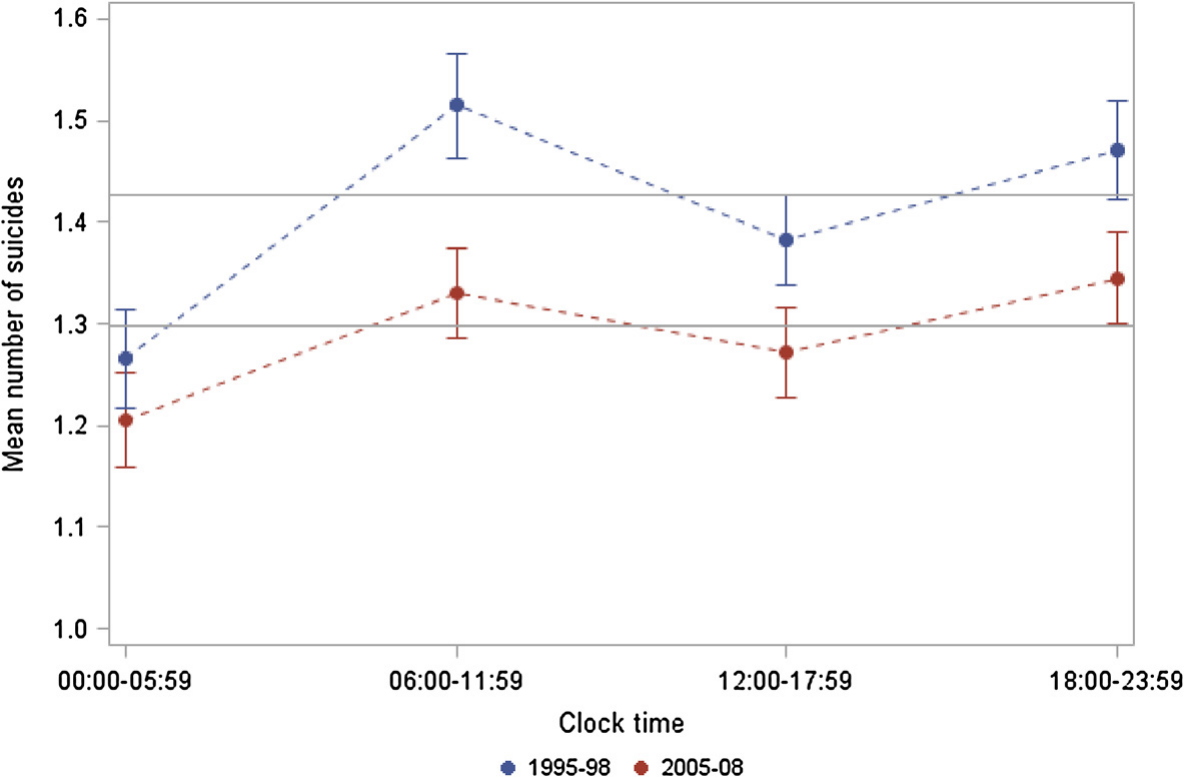
**Mean number of railway suicides per 6 h time period (blue upper line: 1995–1998, red lower line: 2005–2008) with 95% confidence intervals.** The horizontal lines indicate the expected numbers in each period.

In both periods, a higher mean number of suicidal events on the railway net was observed between 6:00 am and 11:59 am (1995–1998: 1.51; 2005–2008: 1.33) and between 6:00 pm and 11:59 pm (1995–1998: 1.47; 2005–2008: 1.35) (Figure 2). This circadian pattern was highly significant in both observation periods (p values < 0.001) and was not substantially affected by the observation period (p value for period effect 0.112), indicating a rather stable pattern over time (data not shown).

### Monthly patterns

**Figure 3:**
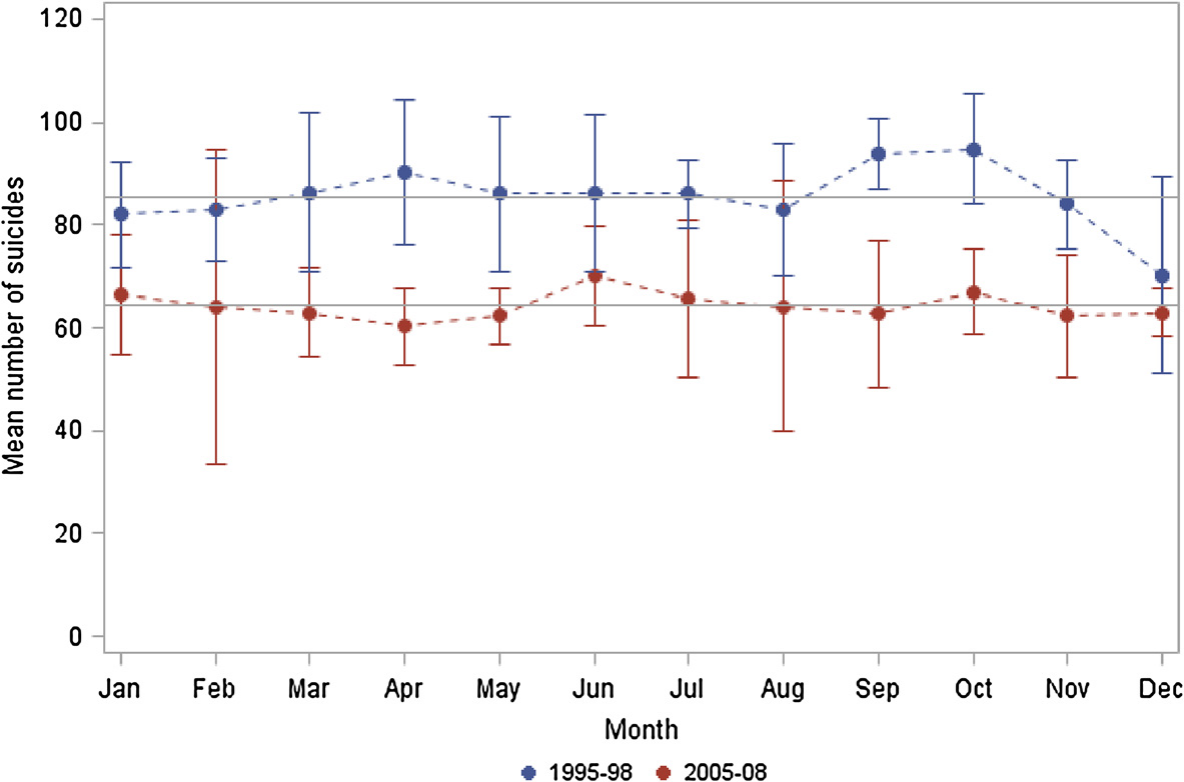
**Mean number of railway suicides per month (blue upper line: 1995–1998, red lower line: 2005–2008) with 95% confidence intervals.** The horizontal lines indicate the expected numbers in each period.

As shown in Figure 3, seasonality was only prominent in the period 1995–1998 (p value 0.018), with higher numbers of railway suicides in September and October (about 94 events on average each year) and lowest numbers in January (70 events on average each year). In contrast, the number of railway suicides was more uniform in the second period (2005–2008) and seasonality could not be observed (p value 0.970). However, the differences in monthly patterns by observation period were not significant (p value for period effect 0.297). When comparing ratios (number of railway suicides in the period 2005–2008 to number of railway suicides in the period 1995–1998), the largest differences among the ratios were observed in April and May as well as in September and October.

## Discussion

The major finding of this study is that weekly and circadian patterns of railway suicide are not statistically different in two observational time periods (1995–1998 vs. 2005–2008), thereby confirming stable behavioural patterns in the long-term.

A bimodal circadian distribution was observed in both periods, with a morning peak between 6:00 am and noon, and an evening/night peak between 6:00 pm and midnight. The weekday distribution followed a similar pattern in both periods, whereby the highest railway suicide frequencies were observed on Mondays and Tuesdays, and the lowest on Saturdays and Sundays. Our results are consistent with previous studies [17,18,21]. The high frequency of railway suicides at the beginning of the week might be explained by factors internal to the railway network, e.g. the volume of services over the week, as well as factors external to the railway network, including a “broken promises” effect that can develop from the elevated sense of expectancy implicitly occasioned by either a positively valued event (weekend) or the threshold of a new cycle (Monday) [22]. The implied promise associated with the arrival of a new temporal cycle is usually not fulfilled and thus, “the stage is set” [22] for a drop in mood accompanied by feelings of personal failure and isolation in high risk subjects [17]. Regarding seasonality, an autumn peak was observed within the period 1995–1998 only. The lack of seasonality in the second observation period (2005–2008) might be explained by preventive measures which were implemented within or shortly after the first observation period. Although previous studies also report a disappearance of seasonal asymmetries towards the end of the 20th century, which was in parts due to the increased prescription of antidepressants [17,23-26], other findings revealed a seasonality of suicides in Finland [27]. This inconsistency might be explained by interplay of factors potentially involved in seasonality (e.g. light conditions, temperature) and by the applied methodology [28,29].

Our findings provide evidence that the seemingly personal choice of a desperate subject to use the railway track as means of suicide might be guided by an underlying mechanism of decision making. The findings of stable time patterns must be included in prevention strategies such as awareness and gatekeeper training, teaching participants to pay increased attention to deviant behaviour, especially at stations, at the identified time windows of high risk, i.e. the beginning of the week and morning and evening hours. Although there is recent evidence that railway suicides are independent of railway density and passenger volume [30], increased awareness should be paid to commuters’ rush hours. Railway and security companies are well advised to meet this challenge by having more staff members at service during the high risk time windows.

As shown in our study, the number of railway suicides was lower in the second period (2005–2008). In general, suicide numbers in Germany declined [1,31], so one may assume that subjects did not turn to other means of suicide. The decline in railway suicides can rather be explained by an array of preventive measures that were implemented in 2002 within the framework of the German Railway Suicide Prevention Project, including an awareness programme, media approaches, hotspot analysis [32] and the introduction of a rule regarding announcements to passengers waiting in station or trains, which requires avoidance of the term “suicide”, and an indication that the delay is due to a “medical rescue operation underway” [3].

The strength of the present study is a large data base covering all suicides on the German railway net by a national registry including the exact time. As a limitation, no valid data on sex and age were available and therefore, no age- and sex-standardizations could be performed.

## Conclusions

The weekday and circadian patterns did not change between the two observation periods, indicating that railway suicide behaviour follows stable temporal patterns. Therefore, these patterns should be an integral part of gatekeeper training programmes. The lack of a pronounced seasonality in the second period might be explained by effective preventive measures.

## Competing interests

The authors declare that they have no competing interests.

## Authors’ contributions

KL drafted the manuscript, and analysed and interpreted the data. JB performed the statistical analyses, interpreted the data and reviewed the manuscript critically. NE helped drafting the manuscript and reviewed it critically. KHL was responsible for the study design and reviewed the manuscript critically. All authors read and approved the final manuscript.

## Pre-publication history

The pre-publication history for this paper can be accessed here:

http://www.biomedcentral.com/1471-2458/14/124/prepub
